# Structural Studies of the Lipopolysaccharide of *Aeromonas veronii* bv. *sobria* Strain K133 Which Represents New Provisional Serogroup PGO1 Prevailing among Mesophilic Aeromonads on Polish Fish Farms

**DOI:** 10.3390/ijms22084272

**Published:** 2021-04-20

**Authors:** Katarzyna Dworaczek, Maria Kurzylewska, Magdalena Laban, Dominika Drzewiecka, Agnieszka Pękala-Safińska, Anna Turska-Szewczuk

**Affiliations:** 1 Department of Genetics and Microbiology, Institute of Biological Sciences, Maria Curie-Skłodowska University, Akademicka 19 St., 20-033 Lublin, Poland; pakiet.kat@gmail.com (K.D.); mariakurzylewska@wp.pl (M.K.); magda.laban@onet.pl (M.L.); 2 Laboratory of General Microbiology, Department of Biology of Bacteria, Faculty of Biology and Environmental Protection, University of Łódź, Banacha 12/16 St., 90-237 Łódź, Poland; dominika.drzewiecka@biol.uni.lodz.pl; 3 Department of Fish Diseases, National Veterinary Research Institute, Partyzantów 57 St., 24-100 Puławy, Poland; a.pekala@piwet.pulawy.pl

**Keywords:** *Aeromonas*, fish pathogen, lipopolysaccharide (LPS), structure elucidation, O-antigen, O-polysaccharide, bacillosamine, d-QuiN4N, NMR spectroscopy, MALDI-TOF mass spectrometry

## Abstract

In the present work, we performed immunochemical studies of LPS, especially the O-specific polysaccharide (O-PS) of *Aeromonas veronii* bv. *sobria* strain K133, which was isolated from the kidney of carp (*Cyprinus carpio* L.) during an outbreak of motile aeromonad infection/motile aeromonad septicemia (MAI/MAS) on a Polish fish farm. The structural characterization of the O-PS, which was obtained by mild acid degradation of the LPS, was performed with chemical methods, MALDI-TOF mass spectrometry, and ^1^H and ^13^C NMR spectroscopy. It was revealed that the O-PS has a unique composition of a linear tetrasaccharide repeating unit and contains a rarely occurring sugar 2,4-diamino-2,4,6-trideoxy-D-glucose (bacillosamine), which may determine the specificity of the serogroup. Western blotting and ELISA confirmed that *A. veronii* bv. *sobria* strain K133 belongs to the new serogroup PGO1, which is one of the most commonly represented immunotypes among carp and trout isolates of *Aeromonas* sp. in Polish aquacultures. Considering the increase in the MAI/MAS incidences and their impact on freshwater species, also with economic importance, and in the absence of an effective immunoprophylaxis, studies of the *Aeromonas* O-antigens are relevant in the light of epidemiological data and monitoring emergent pathogens representing unknown antigenic variants and serotypes.

## 1. Introduction

The world production of farmed food fish relies increasingly on inland aquaculture, which is typically practiced in a freshwater environment in most countries. It is estimated that freshwater species, such as carp, rainbow trout, catfish, and tilapia, are expected to represent about 62% of total world aquaculture production in 2030, compared with 58 percent in 2016 [1,2]. However, a worrying phenomenon is the occurrence of health disorders and infectious diseases in freshwater fish species, which contribute to large economic losses exceeding $10 billion worldwide. Diseases in aquacultures can be caused by various factors, however the most important among bacterial infections are those caused by motile *Aeromonas* representatives [3,4].

Aeromonads are Gram-negative, rod-shaped bacteria occurring ubiquitously in freshwater and marine environments, including drinking-water distribution systems, even when the water supply is chlorinated [5,6,7], and wastewater systems [8,9]. *Aeromonas* bacteria are especially known as opportunistic pathogens of fish. Outbreaks in cultured fish are caused by stressful environmental conditions such as sudden water temperature changes, excessive stocking density, or poor water quality [10]. The infections observed in freshwater fish comprise a wide variety of clinical manifestations, from skin ulceration including gill and fin lesions known as MAI (motile aeromonad infection) to the acute form of the disease, which quickly leads to sepsis, called MAS (motile aeromonad septicemia). In both the MAI and MAS modes of the disease, a high mortality rate of approximately 80% of stock is observed [3].

The pathogenicity of *Aeromonas* is associated with the production and/or secretion of numerous virulence factors, such as aerolysin, haemolysin, enterotoxins, proteases, and hemagglutinins. Moreover, these proteins and enzymes help to distinguish between potentially pathogenic and non-pathogenic strains [11,12]. The mechanisms of iron acquisition known as metallostasis, have also been described as important factors in the development of aeromonad infections [13,14,15]. In addition, components of the Gram-negative bacterial cell envelope, i.e., the capsule, S-layer, lipopolysaccharide (LPS), outer membrane proteins, and structures involved in the colonization process, e.g., polar flagella and pili, play a significant role in the pathogenicity of *Aeromonas* [11,16].

The lipopolysaccharide molecule is a glycolipid and a key trigger of innate immune responses, ranging from local inflammation to disseminated sepsis [17,18]. LPS contains three structural regions: the hydrophobic lipid A (endotoxin), a phosphorylated and nonrepetitive core oligosaccharide (core OS), and an O-specific polysaccharide (O-polysaccharide, O-PS, O-antigen) [19]. The O-polysaccharide is a polymer with highly antigenically variable oligosaccharide repeating subunits (a heteropolymeric variant of the O-antigen) and, according to the structure and composition of this surface polysaccharide, bacteria can be classified into different serotypes and serovars [20], which is important for diagnostics and epidemiological monitoring.

Regarding the *Aeromonas* spp. serotyping, the original classification scheme proposed by Sakazaki and Shimada, which included 44 different O-serogroups (the NIH scheme; National Institute of Health, Japan), was later supplemented by addition of new provisional serogroups, and it currently consists of 97 O-serogroups [21,22]. Serotyping of pathogenic bacteria leads to recognition of the etiological agent associated with specific disease syndrome [23]. Since there are differences in the distribution of the dominant O-serogroups responsible for the onset of various diseases depending on geographical regions [24], as evidenced by epidemiological studies carried out in various areas, including Polish aquacultures indicating to the emergence of new O-antigen variants, there are reasons to extend further or modify the classical serogrouping scheme [25,26,27].

In the present work, we have shown the structural characterization of LPS (including the O-specific polysaccharide) isolated from *A. veronii* bv. *sobria* strain K133, which has been classified into the new provisional serogroup PGO1, i.e., the most commonly represented immunotype among isolates pathogenic to carp and trout in Polish aquacultures. We have demonstrated that the O-chain is composed of a linear tetrasaccharide repeating unit and contains a rarely occurring sugar 2,4-diamino-2,4,6-trideoxy-D-glucose (bacillosamine), which may determine the specificity of this new serogroup. To the best of our knowledge, this is the first paper describing the occurrence of bacillosamine as a component of *Aeromonas* O-antigens. Moreover, the composition and structure of the O-PS repeating unit elucidated in the paper is unique among known bacterial polysaccharides.

## 2. Results

### 2.1. Bacterial Cultivation, Isolation of LPS, and SDS-PAGE Study

**Figure 1:**
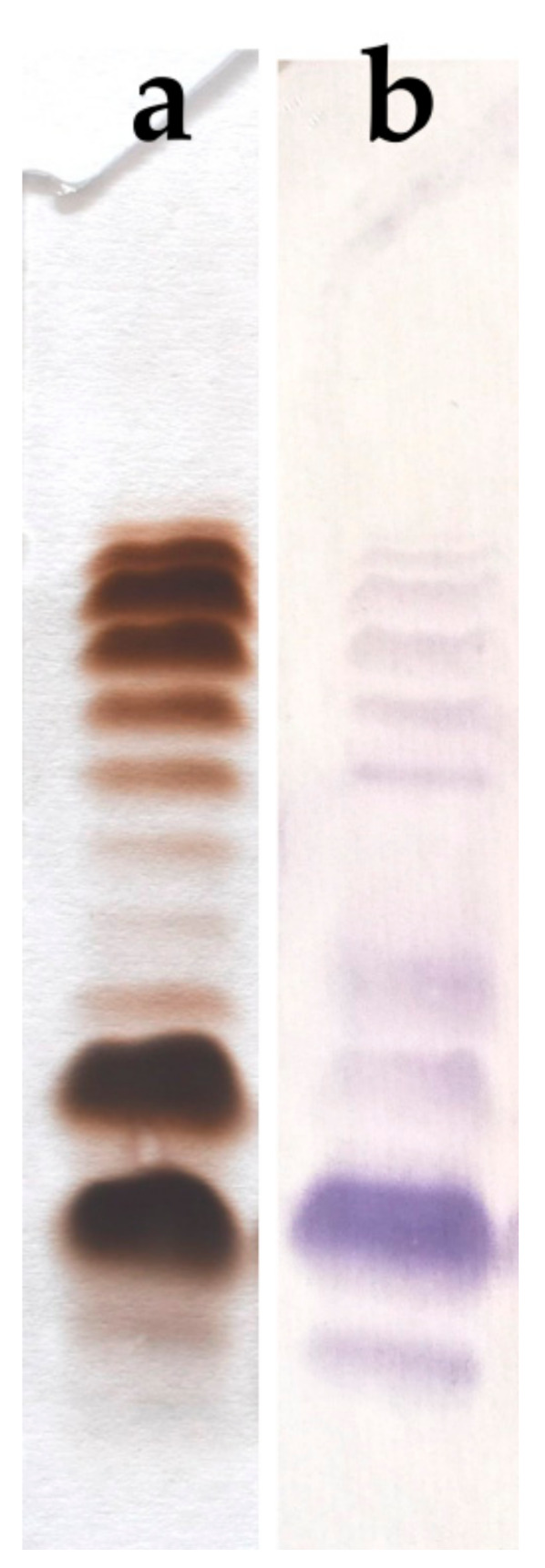
(**a**) Silver-stained SDS-PAGE of phenol-soluble LPS from *A. veronii* bv. *sobria* strain K133 (3 μg were loaded per lane), and (**b**) Western blot with the PGO1 reference antiserum.

*Aeromonas veronii* bv. *sobria* K133 bacteria were cultivated in TSB (tryptic soy broth) at 28 °C for 72 h, and the cell biomass obtained was enzymatically treated, as described in Section 4, and then subjected to lipopolysaccharide extraction using the hot phenol-water method [28]. The HMW (high molecular weight) S-LPS species were recovered only from the phenol phase in a yield of 1.2% of the dry bacterial cell mass. After silver nitrate gel staining, the SDS-PAGE analysis of the phenol-soluble LPS revealed a profile characteristic for glycoforms isolated from smooth bacterial cells composed of both fast-migrating LMW (low molecular weight) species typical of rough R- and semi rough SR-LPS, and slow-migrating species representing S-LPS glycoforms with different chain lengths of the O-antigen polysaccharide (O-PS) (Figure 1a).

### 2.2. Serological Studies of the Aeromonas veronii bv. sobria Strain K133 O-PS

*A. veronii* bv. *sobria* strain K133 was serologically typed by agglutination tests using heat-inactivated bacteria and antisera for 44 defined *Aeromonas* spp. O-serogroups (NIH system) and 20 provisional serogroups (PGO1—PGO20) for selected Polish isolates. Based on these tests, strain K133 was classified into the serogroup PGO1 [27,29]. The affiliation to the serogroup was further confirmed by the reactivity of the PGO1 reference antiserum with the phenol-soluble LPS and with both the whole bacterial cells and the LPS of *A. veronii* bv. *sobria* strain K133 in the Western blotting and ELISA experiments, respectively.

The Western blot with the PGO1 antiserum revealed positive reaction in the region of S-LPS and SR-LPS species (Figure 1b). In addition, the stained bands corresponding to the fast-migrating R-LPS may also indicate the presence of immunoglobulins in the antiserum, which recognize structural determinants in the core oligosaccharide region.

Accordingly, in the ELISA experiment (Table 1), the rabbit polyclonal reference antiserum reacted with whole bacterial cells and the LPS of *A. veronii* bv. *sobria* strain K133 to the titer of 128,000 and 64,000, respectively. However, the reactions were weaker than that in the homologous system with the PGO1 cells. Adsorption of the PGO1 reference antiserum with the *A. veronii* bv. *sobria* K133 cells decreased its reactivity in the homologous system, while there was no reaction of the adsorbed PGO1 antiserum with both the whole bacterial cells and the LPS of *A. veronii* bv. *sobria* K133. The latter findings indicated that the adsorption process was complete and resulted in removal of anti-K133 antibodies from the PGO1 reference antiserum. Moreover, these data also demonstrated that the PGO1 antiserum contained additional immunoglobulins recognizing structural determinants that were not found in the *A. veronii* bv. *sobria* K133 O-antigen.

Therefore, detailed chemical analyses were performed to establish the structure of the O-PS from *A. veronii* bv. *sobria* strain K133, which represents the PGO1 serogroup.

### 2.3. Chemical and Mass Spectrometry Analyses of LPS

The compositional analysis of the degraded polysaccharide (dgPS) fraction released from the phenol-soluble LPS after mild acid hydrolysis was performed using GLC-MS of alditol acetates. The analysis showed the presence of d-glucose (d-Glc), d-galactose (d-Gal), 2-amino-2-deoxy-d-glucose (d-GlcN), 2-amino-2-deoxy-d-galactose (d-GalN), d-*glycero*-d-*manno*-heptose (d,d-Hep), and l-*glycero*-d-*manno*-heptose (l,d-Hep) in a ratio of 2.0:5.0:1.0:6.2:5.3:4.2. Kdo (3-deoxy-d-*manno*-2-octulosonic acid)—the only acidic sugar—was found in the LPS after treatment of the LPS with 48% aqueous HF (hydrofluoric acid), which suggested its phosphorylation [30,31,32]. In addition, the GLC-MS analysis of fatty acids as methyl esters and *O*-TMS derivatives revealed 3-hydroxytetradecanoic C14:0(3-OH), 3-hydroxy*iso*pentadecanoic C*i*15:0(3-OH), dodecanoic C12:0, and tetradecanoic C14:0 acids as the major components in a ratio of 5.5:1.4:2.6:1.0. GlcN was identified as a constituent of the lipid A disaccharide.

**Figure 2:**
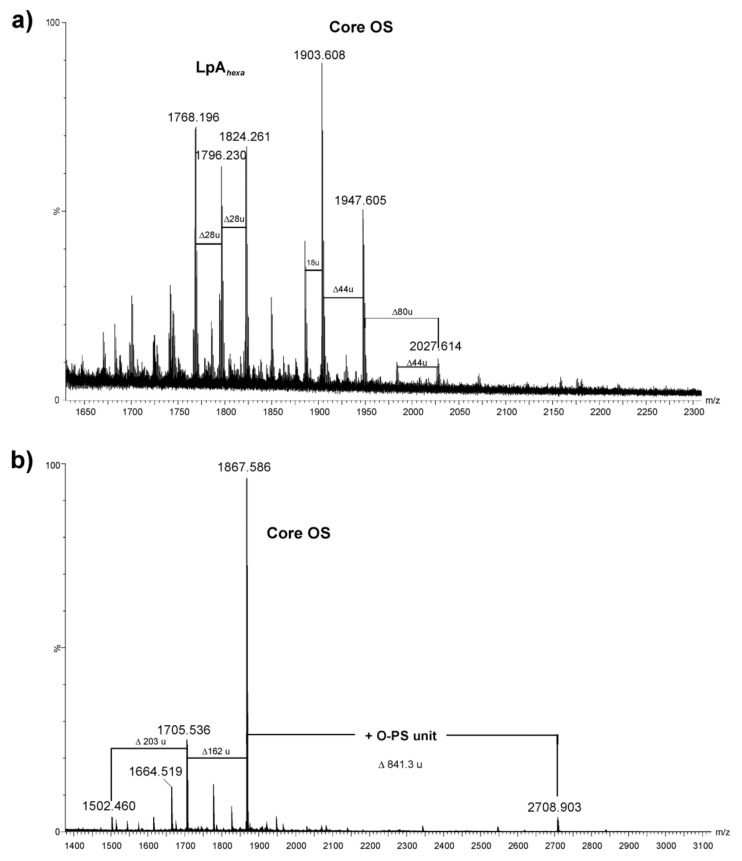
MALDI-TOF mass spectra (negative ion mode) of the LPS (**a**) and low molecular mass fraction (**b**) of *A. veronii* bv. *sobria* strain K133. The notations indicate: 18 u—loss of H_2_O, 28 u—differences in (CH_2_)_2_ in the fatty acid chain length; 44 u—loss of CO_2_; 80 u—loss of phosphate; 162 u—loss of hexose, 203 u—loss of *N*-acetyl hexosamine, LpA*_hexa_*—hexaacylated lipid A; Core OS—core oligosaccharide.

The negative ion (MALDI-TOF) mass spectrum of the *A. veronii* bv. *sobria* K133 lipopolysaccharide (Figure 2a) showed the most intensive signals in the *m*/*z* range 1600–2000, which were attributable to the lipid A and core oligosaccharide species (Y- and B-type fragment ions) arising from in-source fragmentation at the ketosidic bond between the core oligosaccharide and the lipid A [33]. The ions at *m*/*z* 1768.19, 1796.23, and 1824.26 corresponded to hexaacylated lipid A species (Y-fragment ions) [25] composed of a bisphosphorylated glucosaminyl disaccharide and substituted by different chain-length fatty acids. The ion at *m*/*z* 1796.23 represented the lipid A species substituted by three 3-hydroxytetradecanoic acids C14:0(3-OH), one 3-hydroxy*iso*pentadecanoic acid C*i*15:0(3-OH), and two tetradecanoic acids C14:0. In turn, the ion at *m*/*z* 1768.19 contained a sugar backbone acylated by four 3-hydroxytetradecanoic acids and two dodecanoic acids, instead of two tetradecanoic acids, compared with the ion at *m*/*z* 1824.26. The composition of the ions is shown in Table 2.

The signals at *m*/*z* 2027.6, 1947.6, and 1903.6 (B-fragment ions) were assigned to the core oligosaccharide with the following composition: HexNAc_1_HexN_1_Hex_2_Hep_5_Kdo*_anh_*P. The mass difference between the first two ions of 80 amu corresponded to bisphosphorylated and monophosphorylated core oligosaccharides, respectively. In turn, the most abundant ion at *m/z* 1903.6 represented a core decasaccharide with loss of the carboxyl group of Kdo.

The negative ion mass spectrum of the core oligosaccharide fraction (core OS), which was liberated from LPS after mild acid hydrolysis and separation by GPC, showed the major ion at *m/z* 1867.58 corresponding to the dephosphorylated core decasaccharide with the composition HexNAc_1_HexN_1_Hex_2_Hep_5_Kdo*_anh_* (calcd monoisotopic mass = 1868.629 amu, calcd mass of deprotonated molecule = 1867.621 amu) (Figure 2b, Table 2). The proposed structure of the core oligosaccharide corresponded to the compositional analysis of the fraction performed by GLC-MS of alditol acetate derivatives.

In addition, the ion at *m/z* 2708.9, observed at a higher mass range of the MALDI-TOF mass spectrum, was attributed to the core oligosaccharide with one O-antigen repeating unit attached. The mass difference of 841.31 amu, corresponding to the following composition: [6dHexNAcNAcyl_1_HexNAc_2_Hex_1_]-H_2_O (calcd monoisotopic mass: 841.332), was in complete agreement with the structure of the O-PS repeating unit established based on the NMR experiments (see Section 2.4).

### 2.4. Structural Studies of O-Polysaccharide (O-PS)

**Figure 3:**
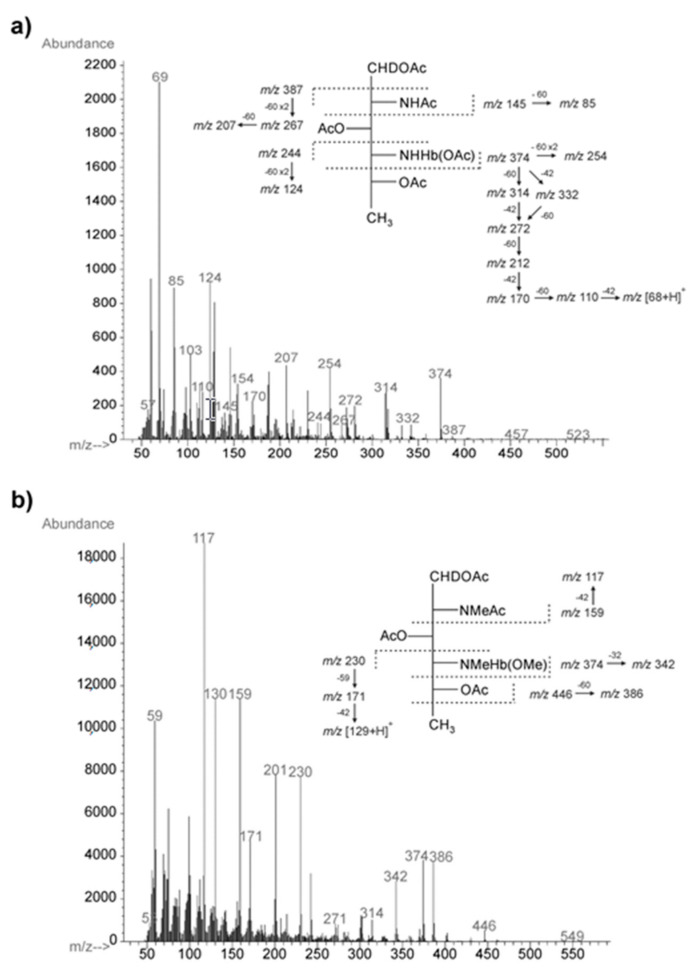
*EI* mass spectra and fragmentation pathways of the alditol acetates of 2,4,6-trideoxy-4-[3-hydroxybutanoylamido]-d-glucose (**a**) and 2,4,6-trideoxy-2-(*N*-methylacetamido)-4-(3-methoxy-*N*-methylbutanoylamido)-d-glucose (**b**) obtained from the O-PS of *A. veronii* bv. *sobria* strain K133. Diagnostic primary and secondary fragment ions are indicated. The mass difference 42, 59, 60, or 32 indicates loss of chetene, acetamide, acetic acid, or methanol, respectively.

The O-PS was released from the phenol-soluble LPS by mild-acid degradation followed by centrifugation of the lipid A precipitate and isolated, in a void volume, by gel-permeation-chromatography (GPC) on a Sephadex G50 Fine column. The yield of the high-molecular-mass O-PS fraction was 22% of the LPS mass. The GLC-MS sugar analysis of alditol acetates obtained after full acid hydrolysis of the O-PS with 2 M CF_3_CO_2_H showed the presence of galactose (Gal) and galactosamine (GalN) as the major components, in a peak area ratio of 1.0:1.6. There were also smaller amounts of glucose, two heptose isomers, and a component with a longer retention time identified as 2,4-diamino-2,4,6-trideoxyhexitol containing a 3-hydroxybutanoyl group (Hb) amido-linked at C-4. The electron impact *EI* mass spectrum (Figure 3a) of the latter compound as an alditol acetate derivative showed ions at *m/z* 145, 244, 374, and 387 for the (C-1 ÷ C-2), (C-4 ÷ C-6), (C-1 ÷ C-4) and (C-2 ÷ C-6) primary fragments, respectively, and the derived secondary fragment ions at *m/z* 85, 124, 254, and 207, which allowed to distinguish the location of the *N*-Hb group.

Moreover, when stronger conditions of the O-polysaccharide hydrolysis (4 M HCl, 100 °C, 16 h) were applied, 2,4-diamino-2,4,6-trideoxyhexitol (identified as QuiN4N, see below) was detected. The latter finding was confirmed by the NMR spectroscopic studies of the O-PS.

The determination of the absolute configuration of the monosaccharides by GLC of acetylated (*S*)-2-octyl glycosides [34] indicated the presence of d-Gal and d-GalN. The d-configuration of QuiN4N (bacillosamine) was inferred from the analysis of glycosylation effects on the ^13^C NMR resonances in the O-PS (see below). The *S* configuration of the Hb group was established by GLC-MS of the *O*-TMS (*S*)-2-octyl ester of 3-hydroxybutanoic acid released by strong acid hydrolysis of the O-PS [35].

The methylation analysis of the O-PS completed the compositional data and resulted in identification of 1,3,5-tri-*O*-acetyl-2,4,6-tri-*O*-methylhexitol-1-*d* (derived from 3-substituted Gal), 1,4,5-tri-*O*-acetyl-2-deoxy-3,6-di-*O*-methyl-2-(*N*-methyl)acetamidohexitol-1-*d* (derived from 4-substituted GalN), 1,3,5-tri-*O*-acetyl-2-deoxy-4,6-di-*O*-methyl-2-(*N*-methyl)acetamidohexitol-1-*d* (derived from 3-substituted GalN), and 1,3,5-tri-*O*-acetyl-2,4,6-trideoxy-2-(*N*-methylacetamido)-4-(3-methoxy-*N*-methylbutanoylamido)hexitol-1-*d* (derived from 3-substituted QuiN4NHb). The electron impact *EI* mass spectrum of the latter derivative contained ions at *m/z* 159, 230, and 374 characteristic of the (C-1 ÷ C2), (C-4 ÷ C6), and (C-1 ÷ C4) primary fragments, respectively, and confirmed the position of the 3-hydroxybutanoyl group at N-4 of QuiN4N (Figure 3b).

The O-polysaccharide structure of *A. veronii* bv. *sobria* strain K133 was then studied with the use of 1D and 2D NMR spectroscopy.

**Figure 4:**
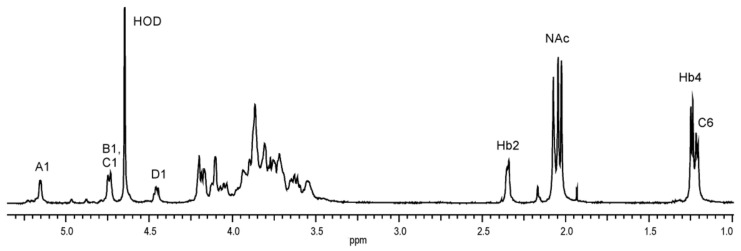
^1^H NMR spectrum of the O-PS of *A. veronii* bv. *sobria* strain K133. The spectrum was recorded in D_2_O at 42 °C at 500 MHz. Capital letters and Arabic numerals refer to atoms in the sugar residues denoted as shown in Table 3. NAc—*N*-acetyl groups.

A low-field region of the ^1^H NMR spectrum (Figure 4) of the O-PS contained signals for anomeric protons at *δ* 5.16 (^3^*J*_1,2_~3.3 Hz), 4.74, and 4.46 (^3^*J*_1,2_~7.5 Hz) with an integral intensity ratio of 1.0:1.8:0.96. However, the signal at *δ* 4.74 corresponded in fact to two overlapping anomeric protons (both ^3^*J*_1,2_~8 Hz), which was demonstrated in the further 2D homonuclear ^1^H,^1^H DQF-COSY, TOCSY, and NOESY experiments.

A high-field region of the spectrum included signals for two C*H_3_*-CH groups: H-6 of QuiN4N and H-4 of Hb at *δ* 1.21–1.25, one CH_2_ group of Hb at *δ* 2.35, and three signals for *N*-acetyl groups at *δ* 2.03, 2.05, and 2.07. Additionally, the ^1^H NMR spectrum showed proton signals of C*H_2_*-OH of hexose and aminohexose residues and C*H*-OH of H-3 of the 3-hydroxybutanoyl group at *δ* 4.20, as well as the ring proton signals in the range of *δ* 3.54–4.21, some of which overlapped.

The analysis of the two dimensional homonuclear (^1^H,^1^H DQF-COSY, TOCSY, and NOESY) and heteronuclear (^1^H,^13^C HSQC, ^1^H,^13^C H2BC, and ^1^H,^13^C HMBC) NMR experiments resulted in the assignment of the ^1^H and ^13^C resonances to the O-PS of *A. veronii* bv. *sobria* strain K133. The ^1^H and ^13^C NMR chemical shifts are collected in Table 3.

**Figure 5:**
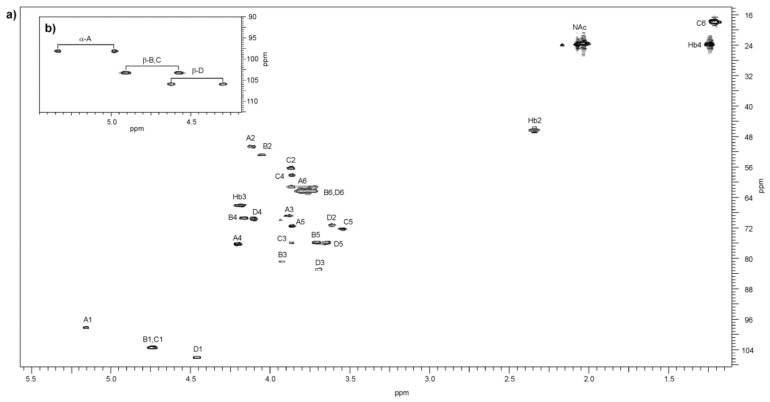
^1^H,^13^C HSQC spectra (500 × 125 MHz) of the O-PS of *A. veronii* bv. *sobria* strain K133. Overlay of (**a**) the ^1^H-detected HSQC spectrum with ^13^C decoupling during acquisition, and (**b**) the anomeric region of the HSQC spectrum measured without decoupling presenting the ^1^*J*_C1,H1_ coupling constant values of α- or β-anomeric configurations of monosaccharides. ^1^*J*_C1,H1_ for A (175 Hz), for B and C (163–167 Hz), and D (163 Hz). Capital letters and Arabic numerals refer to atoms in sugar residues denoted as shown in Table 3.

The ^1^H,^13^C HSQC spectrum (Figure 5) contained four correlation signals at *δ* 4.12/50.6, 4.05/52.8, 3.87/56.2, and 3.87/58.0 of protons at the nitrogen-bearing carbons to the corresponding carbons and showed that the O-PS repeating unit contained *N*-acetamido sugars. Moreover, the absence of signals at the ^13^C coordinate in the region of *δ* 83.0–88.0 characteristics of the furanose ring demonstrated that all the sugars were pyranoses [36].

The ^1^H,^1^H TOCSY and DQF-COSY spectra revealed four spin systems for monosaccharide residues, which were labelled **A**–**D** in the order of the decreasing chemical shifts of their H1/H2 protons, and an additional non-sugar spin system corresponding to the *N*-3-hydroxybutanoyl group (**Hb**).

The correlations in the two dimentional homonuclear ^1^H,^1^H TOCSY, DQF-COSY and NOESY (Supplementary Materials Figure S1) spectra revealed three spin systems for sugar residues having the *galacto* configuration and one monosaccharide with the *gluco* configuration (see below).

**Figure 6:**
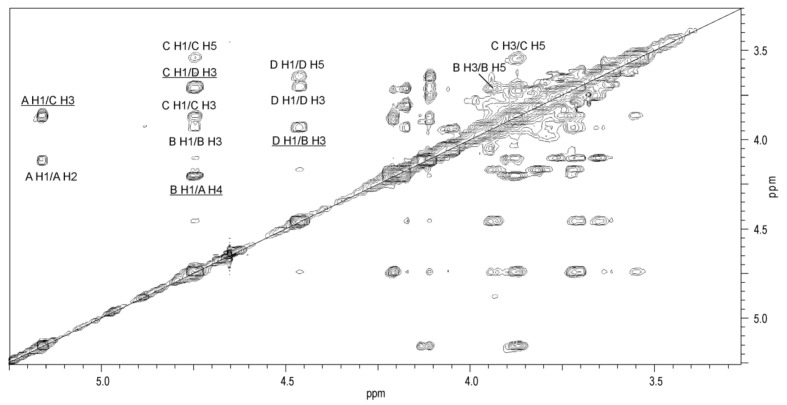
A part of ^1^H,^1^H NOESY spectrum of the O-PS of *A. veronii* bv. *sobria* strain K133. The map shows NOE contacts between anomeric protons and protons at the glycosidic linkages (underlined). Some other H/H correlations are depicted as well. Capital letters and Arabic numerals refer to atoms in the sugars denoted as shown in Table 3.

In the TOCSY spectrum, starting from the H-1 proton signal the correlations with H-2, H-3 and H-4 were visible for spin systems **A**, **B**, and **D**, indicating *galacto*-configured monosaccharides. The remaining resonances were assigned from the NOESY (Figure 6), DQF-COSY and heteronuclear experiments.

In the ^1^H,^13^C HMBC spectrum, correlations of the anomeric proton with carbons C-3 and C-5 were found for the spin system **A**, and then the proton resonances were assigned from the ^1^H,^13^C HSQC spectrum. In the NOESY spectrum, correlations of H-4/H-5 and H-4/H-6 were visible for spin systems **A** and **D**. In turn, for spin system **B**, the chemical shift of H-5 was assigned from the H-3/H-5 and H-4/H-5 intraresidue NOE contacts. The H-6 resonances would then be assigned from H-5/H-6 correlations in the DQF COSY spectrum. The corresponding ^13^C resonances for spin systems **A**, **B**, and **D** were inferred from the ^1^H,^13^C HSQC spectrum. Moreover, in the latter spectrum, the correlations of two H-2 protons at *δ* 4.12 and *δ* 4.05 with the corresponding nitrogen-bearing carbons at *δ* 50.6 and *δ* 52.8, respectively, indicated that two spin systems, i.e., **A** and **B**, were *N*-acetamido sugars. By including *^3^J*_H1,H2_ coupling constant values, spin systems **A**, **B**, and **D** were assigned to α-Gal*p*N, β-Gal*p*N, and β-Gal*p*, respectively.

In the DQF-COSY and TOCSY spectra, correlations of H-1/H-2 up to H-6 typical of monosaccharide having the *gluco* configuration were found for spin system **C**. The corresponding carbons were inferred from the ^1^H,^13^C HSQC spectrum. However, given the almost complete coincidence of the H-2,3,4 proton signals, the chemical shifts of C-2, C-3, and C-4 of spin system **C** were identified after consideration of the two-bond and long-range correlations in the H2BC and HMBC spectra, respectively, and the glycosylation effects on the ^13^C NMR resonances [37].

**Figure 7:**
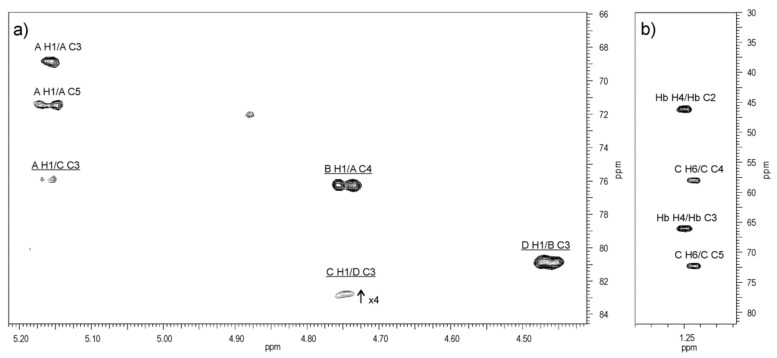
Regions of the ^1^H,^13^C HMBC spectrum of the O-PS of *A. veronii* bv. *sobria* strain K133. The maps show heteronuclear correlations for: (**a**) anomeric protons, and (**b**) H-6 protons. Interresidue correlations between anomeric protons and carbons at the glycosidic linkages are underlined. Some other correlations H/C are depicted as well. Capital letters and Arabic numerals refer to protons or carbons in the sugar residues denoted as shown in Table 3.

The ^1^H,^13^C HSQC spectrum at the ^1^H coordinate (Figure 5a) showed two correlation signals at *δ* 3.87/56.2 and 3.87/58.0 of protons at the nitrogen-bearing carbons to the corresponding carbons, which after including the high-field positions of H-6/C-6 at *δ* 1.21/17.1 indicated that spin system **C** was a diamino-6-deoxysugar. The latter finding was confirmed in the HMBC spectrum (Figure 7) by the presence of a long-range correlation between H-6 and C-4 at *δ* 1.21/58.0, which allowed identifying C-4 as the second nitrogen-bearing carbon. The high-field region of the HMBC spectrum at the ^1^H coordinate of the methyl protons (*δ* 1.21) also showed a correlation to C-5 of residue **C** at *δ* 72.3, whose low field position indicated β-linked sugar [32,38]. Based on these data and after including a relatively large *^3^J*_H1,H2_ coupling constant value of ~8 Hz, spin system **C** was assigned to β-Qui*p*N4N.

The ^13^C resonances of the two nitrogen-bearing carbons of QuiN4N were also verified by the two-bond correlations in the ^1^H,^13^C-H2BC experiment. In the spectrum, for the nitrogen-bearing carbon at *δ* 56.2, there were correlations with the protons at *δ* 3.87 and 4.74 (H-1 of residue **C**). In turn, for the second carbon at *δ* 58.0, a connection was found with signals at *δ* 3.87 and 3.54 (H-5 of residue **C**). These correlations confirmed the chemical shifts of C-2 and C-4 QuiN4N at *δ* 56.2 and 58.0, respectively.

Accordingly, the ^1^H,^13^C-H2BC spectrum also showed correlations for two other nitrogen-bearing carbons of residues **A** and **B** to H-1 and H-3 protons at *δ* 5.16/50.6;3.89/50.6 and at *δ* 4.74/52.8;3.93/52.8, respectively.

The ^1^*J*_C1,H1_ coupling constant values determined from the ^1^H,^13^C HSQC spectrum measured without ^13^C decoupling (Figure 5b) of the O-PS confirmed that one of the monosaccharides with C-1 at *δ* 98.2 (**A**) was α-linked (^1^*J*_C1,H1_ 175.0 Hz) and three others with C-1 at *δ* 103.3 (**B**, **C**) and 106.1 (**D**) were β-linked (^1^*J*_C1,H1_ 163–167 Hz) [39].

The anomeric configuration of the sugar residues was also supported by the NOE contacts of H-1 to H-2 for **A** and H-1 to both, H-3 and H-5 for the other three sugars (**B**, **C**, **D**) and confirmed the α- and β-anomeric configurations of monosaccharides, respectively. These intraresidue cross-peaks were informative especially for spin systems **B** and **C** with overlapping H-1 proton signals.

The low-field displacement of the signals for C-4 of **A** (*δ* 76.3) and C-3 of **B** (*δ* 80.8), **C** (*δ* 75.9), and **D** (*δ* 82.8), compared with their resonances in the corresponding non-substituted monosaccharides, indicated the linkage position in each sugar residue [37,39,40].

The substitution pattern and sequence of the monosaccharides in the O-PS repeating unit was established in the NOESY and HMBC experiments. The ^1^H,^1^H NOESY spectrum (Figure 6) showed interresidue cross-peaks between residues **A**→**C**, **C**→**D**, **D**→**B**, and **B**→**A**. The following strong correlations for the transglycosidic protons were observed: Gal*p*N **A** H-1/Qui*p*N4N **C** H-3 at *δ* 5.16/3.87, Qui*p*N4N **C** H-1/Gal*p* **D** H-3 at *δ* 4.74/3.70, Gal*p* **D** H-1/Gal*p*N **B** H-3 at *δ* 4.46/3.93, and Gal*p*N **B** H-1/ Gal*p*N **A** H-4 at *δ* 4.74/4.21.

The HMBC spectrum (Figure 7) demonstrated the following expected correlations between the anomeric protons and the linkage carbons: Gal*p*N **A** H-1/Qui*p*N4N **C** C-3 at *δ* 5.16/75.9, Qui*p*N4N **C** H-1/Gal*p* **D** C-3 at *δ* 4.74/82.8 (week), Gal*p* **D** H-1/Gal*p*N **B** C-3 at *δ* 4.46/80.8, and Gal*p*N **B** H-1/ Gal*p*N **A** C-4 at *δ* 4.74/76.3.

The ^13^C resonance of the NAc carbonyl signals were inferred from the correlations between the H-2 protons of residues **A** and **B** (*δ* 4.12 and *δ* 4.05) and the corresponding carbons in the HMBC spectrum (*δ* 175.5 and 176.4), and between the latter and the methyl proton signals at *δ* 2.05 and 2.07, respectively.

**Figure 8:**
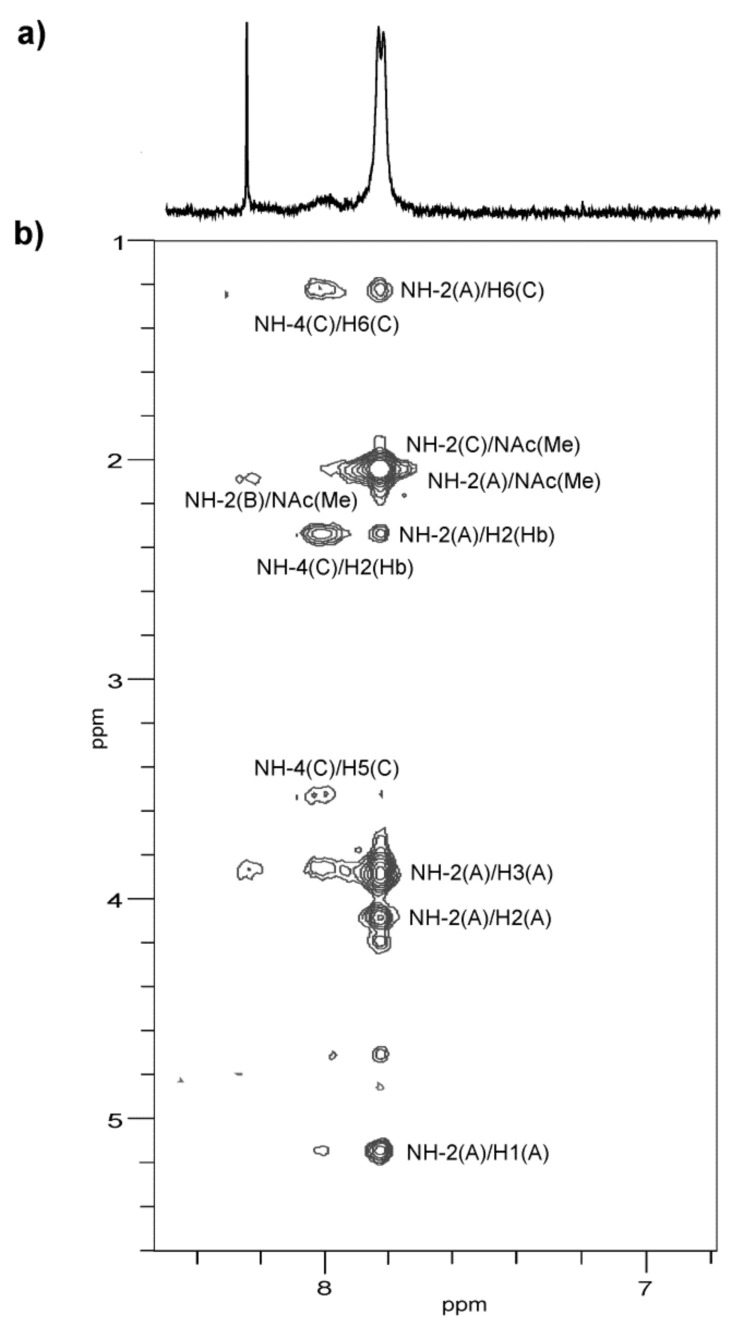
Parts of ^1^H NMR (**a**) and ^1^H,^1^H NOESY (**b**) spectra of the NH region of the O-PS of *A. veronii* bv. *sobria* strain K133 recorded at 20 °C in a 90% H_2_O—10% D_2_O mixture. The map shows NOE contacts for the NH protons. Capital letters and Arabic numerals refer to atoms in the sugars denoted as shown in Table 3.

The distribution of the *N*-acyl (**Hb**) and *N*-acetyl groups was established by the NOESY experiment on the O-PS sample in a 90% H_2_O—10% D_2_O mixture (Figure 8), which enabled detection of nitrogen-linked protons. The ^1^H NMR spectrum revealed three NH protons at *δ* 7.83, 8.02 (a broad signal), and 8.24, which were assigned by a 2D NOESY experiment to NH-2 of both α-GalN (**A**) and QuiN4N (**C**), NH-4 of QuiN4N, and NH-2 of β-GalN (**B**), respectively. The spectrum showed correlations of NH-2 of α-GalN (**A**) and NH-2 of QuiN4N with the NAc methyl protons at *δ* 7.83/2.03–2.05, NH-4 of QuiN4N with H-2 of **Hb** at *δ* 8.02/2.35, and NH-2 of β-GalN (**B**) with the NAc methyl protons at *δ* 8.24/2.07, respectively. Moreover, the intraresidue NOE contacts between NH-4 of QuiN4N and both H-5, H-6 at *δ* 8.02/3.54, 1.21, and the interresidue NOE correlations for **A**(1→3)**C** between NH-2 of α-GalN (**A**) and H-2 (**Hb**)**,** and H-6 of QuiN4N at *δ* 7.83/2.35 and 7.83/1.21, respectively, resulting from the spatial proximity of the residues, confirmed the N-acylation of QuiN4N at position 4.

The d configuration of Qui*p*N4N in the O-PS of *A. veronii* bv. *sobria* strain K133 was established by the analysis of the glycosylation effects on the ^13^C NMR chemical shifts [37,41,42]. In the disaccharide fragment **C**-(1→3)-**D**, β-Qui*p*N4N-(1→3)-β-d-Gal*p*, the large positive α-effect on C-1 of residue **C** (+7.45 ppm) and C-3 of residue **D** (+9 ppm) and the small negative β-effect (−0.1 ppm) on C-4 of **D** indicated that the linked monosaccharides have the same d absolute configuration. In the case of different l-d absolute configurations, the positive α-effect on C-1 of residue **C** and C-3 of residue **D** would have been <4 ppm and <7 ppm, respectively, and the negative β-effect on C-4 of **D** would have been much higher (−3.0 ppm) [37,41].

In the disaccharide fragment **A**-(1→3)-**C,** α-d-Gal*p*NAc-(1→3)-β-Qui*p*N4N, the small β-effect of glycosylation on C-4 of β-Qui*p*N4N **C** (<0.5 ppm) indicated that the linked monosaccharides had the same d absolute configuration. In the case of different d-l absolute configurations, the higher negative β-effect of ~1.4 ppm on C-4 would have been observed [41,42].

Based on the data obtained, it was concluded that the O-polysaccharide from the LPS of *A. veronii* bv. *sobria* strain K133 is composed of a tetrasaccharide repeating unit and has the structure presented below: 
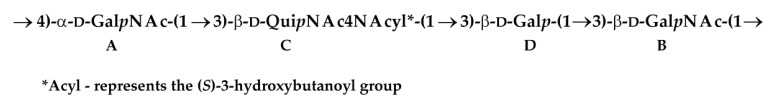


To our knowledge, the O-PS from *A. veronii* bv. *sobria* strain K133 is unique among bacterial polysaccharide structures as indicated by the Bacterial Carbohydrate Structure Database (http://glyco.ac.ru/bcsdb, (accessed on 10 March 2021)) [43].

## 3. Discussion

The inland aquaculture in Poland is focused on two fish species, i.e., carp and rainbow trout [44], the farming of which is endangered due to the stressful environmental conditions and various diseases, with dominance of infections caused by motile *Aeromonas* bacteria [3,4]. Studies on the occurrence of mesophilic *Aeromonas* species associated with outbreaks of MAI/MAS in Polish culture facilities showed the *A. veronii* bv. *sobria* species as one of the dominant isolates on carp farms, while representatives of *A. veronii* bv. *sobria*, *A. bestiarum*, and *A. salmonicida* were pathogenic for both carp and trout. Moreover, the veterinary data indicated that, despite their large diversity, only some *Aeromonas* sp. serogroups seem to be associated with virulence for freshwater fish species [26]. As demonstrated recently, the majority of isolates pathogenic to carp and trout in Polish cultures were positively classified based on the somatic O-antigen variants when the 44 antisera of the NIH scheme were extended to include those for 20 new provisional serogroups of local origin. Moreover, it was evidenced that the highest number of *Aeromonas* strains isolated from carp and trout represented the serogroup PGO1 [27].

Here, we established the structure of the LPS and the O-specific polysaccharide from *A. veronii* bv. *sobria* strain K133, which was isolated from the kidney of carp during an outbreak of MAI/MAS on a Polish fish farm. Strain K133 was classified to the serogroup PGO1, i.e., an immunotype that is common among aeromonads with pathogenicity to fish in Polish aquacultures.

The chemical and MALDI-TOF mass spectrometry analyses have revealed that the LPS of *A. veronii* bv. *sobria* strain K133 contains hexaacylated lipid A species with a conserved architecture of the backbone composed of a 1,4′-bisphosphorylated-β-(1→6)-linked-d-GlcN disaccharide, acylated by three or four residues of 3-hydroxytetradecanoic acids C14:0(3-OH) and saturated fatty acids, i.e., dodecanoic or tetradecanoic acids.

The sugar analysis of the core oligosaccharide revealed d,d-heptose and l,d-heptose residues indicating the type of the core OS shared by *A. hydrophila* [30,31,45] and *A. bestiarum* species [46]. This LPS core variant is different from those described for *A. salmonicida* subsp. *salmonicida*, in which there was only one heptose isomer (l,d-Hep) [47]. However, some differences were found in the structure of the core region studied here. The chemical and mass spectrometry experiments revealed the following composition of the core oligosaccharide: HexNAc_1_HexN_1_Hex_2_Hep_5_Kdo*_anh_*P_1_, whose structure slightly differs from those described for *A. hydrophila* and *A. bestiarum* species by the presence of five instead of six heptose residues and an additional *N*-acetamido hexose (compare with the composition of the core OS of *A. hydrophila* HexN_1_Hex_2_Hep_6_Kdo_1_P_1_).

The SDS-PAGE analysis confirmed that the phenol-soluble LPS contained HMW S-LPS glycoforms and thus suggested a highly hydrophobic character of the O-polysaccharide chains. This finding was in agreement with structural analysis, which demonstrated the presence in the O-PS of deoxy-amino sugars and amino sugars with hydrophobic substituents i.e., *N*-acetyl and *N*-acyl groups.

The Western blotting and ELISA experiments supported the results of serotyping with the use of the agglutination test showing the classification of the strain K133 to the new provisional serogroup PGO1. As reported recently, using heat-inactivated bacteria and antisera for 44 defined *Aeromonas* O-serogroups of the NIH system and after complementing the classical scheme with 20 new antisera for provisional serogroups (PGO1—PGO20) of local origin, the positive classification of the *Aeromonas* sp. isolates based on appropriate somatic antigen increased from 53% (with the use of the NIH system) to nearly 90% after including the new antisera [27,29].

The structure of the O-PS of *A. veronii* bv. *sobria* strain K133 has been established and it has been found that the O-antigen of the strain is built up of linear tetrasaccharide repeating units. To the best of our knowledge, the composition of the O-PS is unique among O-chains of *Aeromonas* spp. and other bacterial polysaccharide structures (Bacterial Carbohydrate Structure Database: http://glyco.ac.ru/bcsdb, (accessed on 10 March 2021)) [43].

While both d-Gal*p* and d-Gal*p*NAc are commonly known as compounds building bacterial polysaccharides, the presence of 2,4-diamino-2,4,6-trideoxy-glucopyranose (bacillosamine) is still a peculiar feature. This sugar has been reported as a component of heteropolymeric O-chains of several bacteria, e.g., *Pseudomonas fluorescens* biovar B strain IMV 247 [48], *Pseudoalteromonas haloplanktis* strain ATCC 14393 [40], *Acinetobacter haemolyticus* strains 57 and 61 [49], *Vibrio cholerae* O5 [50], *V. cholerae* O8 [38], *V. cholerae* O100 [51], *Shewanella japonica* KMM 3299^T^ [52], *Plesiomonas shigelloides* strain 302-73, serotype O1 [53], *Pseudomonas chlororaphis* subsp. *aureofaciens* UCM B-306 [54], and *Idiomarina abyssalis* KMM 227^T^ [55], as well as capsular polysaccharides of *Psychrobacter maritimus* 3pS [56], *Providencia rustigianii* O11 [57], and *Acinetobacter baumannii* clinical isolate MG1 [58]. Additionally, in *Vibrio anguillarum* serotype O2, which affects salmonids and other marine fish species causing vibriosis, bacillosamine has been established as a component of tetrasaccharide repeating units of both the O-chain polysaccharide and acidic capsular polysaccharide [59].

Structural studies have indicated that QuiN4N in microbial polysaccharides usually carries *N*-acetyl groups located at both N-2 and N-4. Such a derivative of diamino-6-deoxyglucose, i.e., *N*,*N*′-diacetylbacillosamine, has been found in the O-PS repeating units of *V. cholerae* O5 and O8 [38,50], *V. anguillarum* O2 [59], *P. haloplanktis* [40], *S. japonica* KMM 3299^T^ [52], *P. chlororaphis* subsp. *aureofaciens* UCM B-306 [54], and *I. abyssalis* KMM 227^T^ [55]. As often as mentioned above, a bacillosamine residue substituted with an *N*-acetyl group at N-2 and a (S)-3-hydroxybutanoyl group at N-4 has been reported. This derivative was found as a component of the O-polysaccharides of *P. fluorescens* strain IMV 247 [48] and *P. shigelloides* strain 302-73 [32], as well as capsular polysaccharides of *A. baumannii* [58], *P. rustigianii* O11 [57], and *P. maritimus* 3pS [56]. In turn, a very unique derivative of QuiN4N with the presence of (*R*)-3,5-dihydroxyhexanoyl group N-linked at position 4 has been indicated only in two O-antigens of *V. cholerae* O3 and *V. cholerae* O100 [51,60].

The ELISA experiment with the reference PGO1 antiserum, carried out in this study, revealed the positive recognition of surface antigenic elements within the whole bacterial cells and the isolated LPS molecules of *A. veronii* bv. *sobria* strain K133, and thus confirmed affiliation of the strain to the PGO1 serogroup. Moreover, the assay also demonstrated that the reference antiserum contained immunoglobulins recognizing a wider range of structural epitopes that were not found in the *A. veronii* bv. *sobria* K133 O-polysaccharide, suggesting both similarities and possible differences in the PGO1 and K133 O antigens. It is worth mentioning, that the O-PS structure of the *Aeromonas* sp. reference strain for the PGO1 serogroup remains unknown. The structural fragment of the O-PS of *A. veronii* bv. *sobria* strain K133, containing β1→3-linked 2-acetamido-4-[(*S*)-3-hydroxybutanoyl]amino-6-deoxyglucose, seems to be one of the key antigenic determinants of the immunospecificity of the PGO1 serotype.

In conclusion, the immunochemical studies of the LPS of *Aeromonas* spp. bacteria, which are pathogenic to freshwater fish species and represent the dominant serogroups, will contribute to advancement in research targeted at development of an effective vaccine based on the antigenic profile of emergent pathogens and dedicated to specific fish farms as an alternative to antibiotic therapy. In fisheries, where bacterial diseases appear systematically and may bring large losses, immunoprophylaxis consisting of the use of preparations that increase non-specific and/or specific immunity in fish should play a significant role in preventing bacterial diseases. Due to the emerging difficulties of effective prophylaxis caused by the lack of commercial vaccines, the interest in the use of autovaccinations that can offer protection against defined serotypes from a specific region or geographic area is increasing in veterinary fields, including ichthyopathology [4].

## 4. Materials and Methods

### 4.1. Bacterial Strain, Growth Conditions, and LPS Isolation

*A. veronii* bv. *sobria* strain K133, serogroup PGO1, was isolated from the kidney of a common carp during an outbreak of MAS/MAI on a Polish fish farm and classified to the species level by restriction analysis of 16S rDNA amplified by PCR [27]. For the LPS studies, strain K133 was obtained from the collection of the Department of Fish Diseases, National Veterinary Research Institute (Puławy, Poland). The bacterium was cultivated with shaking (120 rpm) on tryptic soy broth (TSB) for 72 h at 28 °C. The cells were harvested by low speed centrifugation (8000× *g*, 20 min). The recovered bacterial cell pellet was washed twice with 0.85% saline and once more with distilled water.

The bacterial cells (5 g dry mass) were suspended in 50 mM phosphate buffer (pH 7.0) containing 5 mM MgCl_2_ and treated with lysozyme, RNAse, and DNAse (16 h, 0.6 mg/g) and then with Proteinase K (16 h, 0.6 mg/g). The enzymatically digested biomass was extracted three times with aqueous 45% phenol at 70 °C [35]. Layers separated by centrifugation (3000× *g*, 45 min, 4 °C) were dialyzed against tap and distilled water. LPS species recovered from the phenol and water layers were purified by ultracentrifugation at 105,000× *g* (4 h, 18 °C) and freeze-dried to give a yield of 3% of dry bacterial cell mass.

### 4.2. SDS-PAGE

The phenol-soluble LPS fraction (3 µg) of *A. veronii* bv. *sobria* strain K133 prepared in the sample buffer (2% SDS and 50 mM Tris/HCl (pH 6.8), 25% glycerol, 0.1% bromophenol blue) was separated in 12.5% SDS-Tricine polyacrylamide electrophoresis gel and the profile was visualized by staining with silver nitrate after oxidation with periodate according to the published method [61].

### 4.3. Serological Studies

Western blotting with the rabbit antiserum PGO1 was performed after transferring SDS-PAGE-separated phenol-soluble LPS of *A. veronii* bv. *sobria* strain K133 to Immobilon P (Millipore, St. Louis, MO, USA). The primary antibodies were detected using alkaline phosphatase-conjugated goat anti-rabbit antibodies (Sigma, St. Louis, MO, USA). The blot was developed with nitroblue tetrazolium and 5-bromo-4-chloro-3-indolylphosphate toluidine (Sigma) for 5 min, as described elsewhere [25]. Polyclonal rabbit O-antiserum against the *Aeromonas* sp. reference strain for the provisional serogroup PGO1 was the kind gift from Professor Alicja Kozińska (the National Veterinary Research Institute, Puławy, Poland) [29].

The enzyme-linked immunosorbent assay (ELISA) was performed as described previously [62] with some modifications. In short: 1–2 μg of the *A. veronii* bv. *sobria* strain K133 LPS or 10–20 μg of whole cell biomasses of *A. veronii* bv. *sobria* strain K133 or the *Aeromonas* sp. PGO1 reference strain per well were coated on flat-bottom F96 Maxisorp Nunc-Immuno plates (Thermo Fisher Scientific, Roskilde, Denmark); polyclonal rabbit PGO1 antiserum and rabbit-IgG specific peroxidase-conjugated goat antibodies (Jackson ImmunoResearch, West Grove, PA, USA) were used. 2,2′-Azino-bis(3-ethylbenzothiazoline-6-sulfonic acid) diammonium salt (ABTS) was used as a substrate for peroxidase; the absorbance (A_405_) was measured with the help of a Multiskan Go microplate reader (Thermo Fisher Scientific USA, Vantaa, Finland). The reference PGO1 antiserum diluted 1:50 in PBS (phosphate-buffered saline) was adsorbed on K133 cells during 0.5 h incubation on ice, in the ratio of 100 µL of wet biomass to 1 mL of the serum. The cells were removed by centrifugation and the process was repeated two more times [63].

### 4.4. Degradation of LPS and Isolation of O-Polysaccharide

The phenol-soluble LPS sample (100 mg) was heated in aq 2% acetic acid at 100 °C for 3 h, and the lipid A precipitate was removed by centrifugation (13,000× *g*, 30 min). The supernatant was concentrated and then fractionated by GPC on a column (1.8 × 80 cm) of Sephadex G-50 Fine (Pharmacia, Sweden) using 1% acetic acid as the eluent and monitoring with a differential refractometer (Knauer, Berlin, Germany). The yield of the O-PS fraction was 22% of the LPS portion subjected to hydrolysis.

### 4.5. Chemical Analyses

For neutral and amino sugar analysis, the degraded polysaccharide (dgPS) fraction released from the phenol-soluble LPS after mild acid hydrolysis, the O-PS, and the core oligosaccharide samples were hydrolyzed with 2 M CF_3_CO_2_H (120 °C, 2 h), reduced with NaBD_4_, and peracetylated with a 1:1 (*v*/*v*) mixture of acetic anhydride and pyridine (85 °C, 0.5 h). The O-PS was also hydrolyzed with 4 M HCl for 16 h at 100 °C to release QuiN4N, *N*-acetylated, reduced with NaBD_4_, and peracetylated.

To release acidic sugar (Kdo), LPS was dephosphorylated with 48% aqueous HF (4 °C, 18 h) and dried under vacuum over sodium hydroxide [32]. Methanolysis was performed with 1 M MeOH/HCl (85 °C, 1 h), and the sample was extracted with hexane. The methanol layer was concentrated and the residue was dried and peracetylated.

The absolute configuration of the monosaccharides was determined by GLC of acetylated (*S*)-(+)-2-octyl glycosides using authentic sugars as standards according to a published method [34], except for QuiN4N, whose configuration was determined upon analysis of glycosylation effects on ^13^C resonances in the O-PS.

The absolute configuration of 3-hydroxybutanoic acid (Hb) was determined according to the method of Kenne, et al. [35] with some modifications: after hydrolysis of the O-PS with 2 M CF_3_CO_2_H (120 °C, 4 h), the product was extracted three times with EtOAc, evaporated under nitrogen, and subjected to solvolysis with 2 M HCl in *S*(+)-2-octanol at 80 °C for 16 h. The sample was concentrated to dryness, and trimethylsilylated derivatives were analyzed by GLC-MS and compared with the retention time of *O*-TMS (*S*)-2-octyl esters of authentic (*S*)- and (*R*)-3-hydroxybutanoates as references.

Methylation of the O-PS (1.0 mg) was carried out with methyl iodide in dimethyl sulfoxide in the presence of powdered sodium hydroxide as described by Ciucanu and Kerek [64]. The products were recovered by extraction with chloroform/water (1:1, *v*/*v*), N-acetylated, hydrolyzed with 2 M CF_3_CO_2_H (120 °C, 2 h), N-acetylated, reduced with NaBD_4_ and peracetylated. The partially methylated alditol acetates derivatives were analyzed by GLC-MS.

For fatty acid analysis, a lipid A sample (1 mg) was subjected to methanolysis in 2 M methanolic HCl (85 °C, 12 h). The resulting fatty acid methyl esters were extracted with hexane and converted to their *O*-trimethylsilyl (*O*-TMS) derivatives, as described elsewhere [53,65]. The methanol layer containing methyl glycosides was dried and peracetylated with a pyridine-acetic anhydride mixture. The fatty acid derivatives and acetylated methyl glycosides were analyzed by GLC-MS as above.

All the sample derivatives were analyzed on an Agilent Technologies 7890A gas chromatograph (Agilent Technologies, Wilmington, DE, USA) connected to a 5975C MSD detector (inert XL EI/CI, Agilent Technologies, Wilmington, DE, USA). The chromatograph was equipped with an HP-5MS capillary column (Agilent Technologies, 30 m × 0.25 mm, flow rate of 1 mL/min, He as carrier gas). The temperature program for all the derivatives was as follows: 150 °C for 5 min, then 150 to 310 °C at 5 °C/min, and the final temperature was maintained for 10 min.

### 4.6. NMR Spectroscopy

The O-PS sample (7 mg) was deuterium-exchanged by freeze-drying from a 99.95% D_2_O solution and examined in 99.98% D_2_O. 1D and 2D NMR spectra were recorded at 42 °C on a 500 MHz NMR Varian Unity Inova instrument and calibrated with external acetone (*δ*_H_ 2.225, *δ*_C_ 31.45). Additionally, for detection of nitrogen-linked protons, ^1^H and ^1^H,^1^H NOESY spectra in a 90% H_2_O—10% D_2_O mixture were recorded at 20 °C on a 500 MHz NMR Varian instrument. Standard Varian software (Vnmrj V. 4.2 rev.) was used to acquire and process the NMR data. The homonuclear and heteronuclear two-dimensional experiments: ^1^H,^1^H DQF-COSY, ^1^H,^1^H TOCSY, ^1^H,^1^H NOESY, ^1^H,^13^C HSQC, ^1^H,^13^C H2BC, and ^1^H,^13^C HMBC were conducted for signal assignments and determination of the sugar sequence in the repeating unit. The mixing time of 90 and 200 ms was used in the TOCSY and NOESY experiments, respectively. The ^1^H,^13^C HSQC experiment (gHSQCAD) with CRISIS based multiplicity editing was optimized for a coupling constant of 146 Hz. The ^1^H,^13^C HSQC spectrum (band-selective gHSQCAD) measured without ^13^C decoupling was used to determine the ^1^*J*_C1,H1_ coupling constants for the anomeric carbons. The heteronuclear multiple-bond correlation (HMBC) experiment was optimized for *J*_C,H_ = 8 Hz, with 2-step low-pass filter 130 and 165 Hz to suppress one-bond correlations.

### 4.7. MALDI-TOF Mass Spectrometry (MS)

The LPS and oligosaccharide samples were analyzed with matrix-assisted laser desorption/ionization time-of flight (MALDI-TOF) mass spectrometry (MS) using a Waters SYNAPT G2-*Si* HDMS instrument (Waters Corporation, Milford, MA, USA) equipped with a 1 kHz Nd:YAG laser system. Acquisition of the data was performed using MassLynx software version 4.1 SCN916 (Waters Corporation, Wilmslow, UK). Mass spectra were assigned with a multi-point external calibration using red phosphorous (Sigma) and recorded in the negative ion mode. Phenol-soluble LPS and oligosaccharide samples (both at a concentration of 15 µg/µL) were suspended in a water/methanol (1:1, *v*/*v*) solution (containing 2 mM EDTA for the LPS sample) and dissolved by ultrasonication. After desalting with the use of cation exchange beads (Dowex 50WX8-200; Sigma), one microliter of each sample was transferred onto a well plate covered with a thin matrix film and allowed to dry at room temperature. The matrix solution was prepared from 2′,4′,6′-trihydroxyacetophenone (THAP) (200 mg/mL in methanol) mixed with nitrocellulose (15 mg/mL) suspended in 2-propanol/acetone (1:1, *v*/*v*) in a proportion of 4:1 (*v*/*v*), according to the published method [65,66].

## Figures and Tables

**Table 1:** Reactivity (reciprocal titers) of the PGO1 antiserum (intact or adsorbed) with the LPS of *A. veronii* bv. *sobria* K133 and the cells of both K133 and PGO1 reference strains.

Type of PGO1 Antiserum	PGO1 Cells	K133 Cells	K133 LPS
intact	512,000	128,000	64,000
adsorbed on K133 cells	64,000	<1000	<1000

**Table 2:** Composition of the main species present in the negative ion MALDI-TOF mass spectrum of the LPS of *A. veronii* bv. *sobria* strain K133.

Observed Mass [M − H]^−^	Calculated Mass [M − H]^−^	Monoisotopic Mass [M]	Composition
1768.196	1768.181	1769.188	HexN_2_P_2_[14:0(3-OH)]_4_(12:0)_2_
1796.230	1796.139	1797.146	HexN_2_P_2_[14:0(3-OH)]_3_[*i*15:0(3-OH)](12:0)_2_
1824.261	1824.243	1825.250	HexN_2_P_2_[14:0(3-OH)]_4_(14:0)_2_
1903.608	1903.598	1904.605	[HexNAc_1_HexN_1_Hex_2_Hep_5_Kdo*_anh_*P]-COO
1947.605	1947.588	1948.595	HexNAc_1_HexN_1_Hex_2_Hep_5_Kdo*_anh_*P
2027.614	2027.554	2028.561	HexNAc_1_HexN_1_Hex_2_Hep_5_Kdo*_anh_*P_2_
1705.536	1705.568	1706.576	HexNAc_1_HexN_1_Hex_1_Hep_5_Kdo*_anh_*
1867.586	1867.621	1868.629	HexNAc_1_HexN_1_Hex_2_Hep_5_Kdo*_anh_*
2708.903	2708.953	2709.961	6dHexNAcNAcyl_1_HexNAc_3_HexN_1_Hex_3_Hep_5_Kdo*_anh_*

**Table 3:** ^1^H (500 MHz) and ^13^C NMR (125 MHz) data (*δ*, ppm) for the O-PS of *A. veronii* bv. *sobria* strain K133.

Residue	Chemical Shifts (*δ*, ppm)							
		H-1 C-1	H-2 C-2	H-3 C-3	H-4 C-4	H-5 C-5	H-6 C-6	NAc
→4)-α-d-Gal*p*NAc-(1→	**A**	5.16 98.2	4.12 50.6	3.89 68.8	4.21 76.3	3.87 71.4	3.73; 3.87 61.3	2.05 23.7; 175.5
→3)-β-d-Gal*p*NAc-(1→	**B**	4.74 103.3	4.05 52.8	3.93 80.8	4.17 69.5	3.71 75.9	3.76; 3.81 62.3	2.07 23.7; 176.4
→3)-β-d-Qui*p*NAc4NAcyl-(1→	**C**	4.74 103.3	3.87 56.2	3.87 75.9	3.87 58.0	3.54 72.3	1.21 17.7	2.03 23.7; 176.4
→3)-β-d-Gal*p*-(1→	**D**	4.46 106.1	3.61 71.2	3.70 82.8	4.10 69.6	3.65 75.9	3.76; 3.81 62.3	
(*S*)-3-hydroxybutanoyl	**Hb**	175.0	2.35 46.2	4.20 66.0	1.25 23.7			

## Data Availability

The data presented in this study have been disclosed in the main text and the Supplementary Materials.

## Supplementary Materials

The following are available online at https://www.mdpi.com/article/10.3390/ijms22084272/s1, Figure S1. ^1^H,^1^H NOESY (500 MHz) spectrum of the O-PS of *A. veronii* bv. *sobria* strain K133.
