# Supplementary material for: Structural Studies of the Lipopolysaccharide of Aeromonas veronii bv. sobria Strain K133 Which Represents New Provisional Serogroup PGO1 Prevailing among Mesophilic Aeromonads on Polish Fish Farms
Source: Int J Mol Sci. 2021 Apr 20;22(8):4272. doi: 10.3390/ijms22084272 (PMC8074265; doi:10.3390/ijms22084272)
Supplement: Supplementary file 1 [file ijms-22-04272-s001.zip › ijms-1186497-supplementary.pdf]

Supplementary Materials:

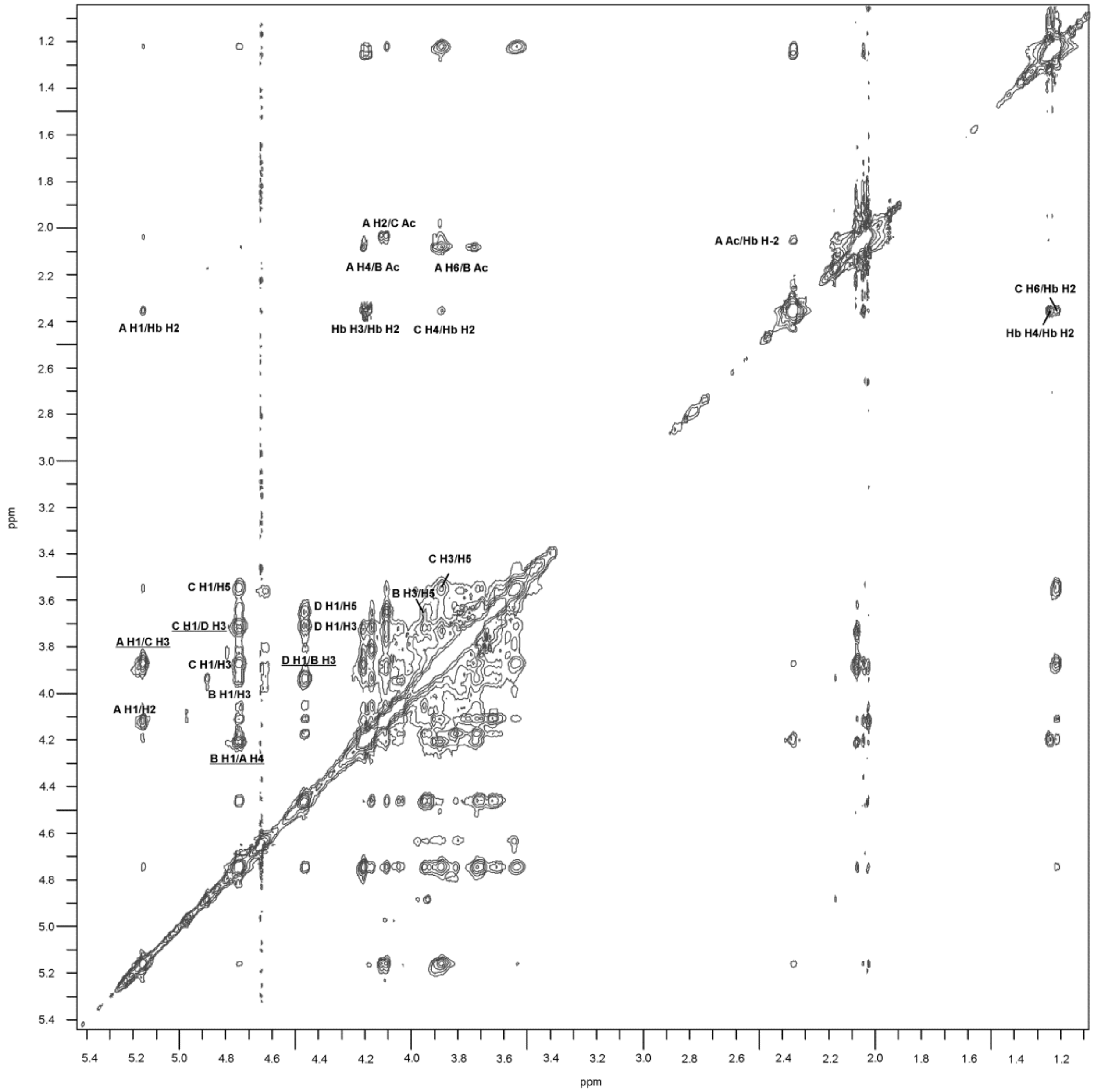

Figure S1: $^{1}H,^{1}H$ NOESY (500 MHz) spectrum of the O-PS of *A. veronii* bv. *sobria* strain K133. The map shows NOE contacts between anomeric protons and protons at the glycosidic linkages (underlined). Some other intraresidue and interresidue correlations are depicted as well. Capital letters and Arabic numerals refer to atoms in the sugars denoted as shown in Table 3.
